# Cinnamic Acid Compounds (*p*-Coumaric, Ferulic, and *p*-Methoxycinnamic Acid) as Effective Antibacterial Agents Against Colistin-Resistant *Acinetobacter baumannii*

**DOI:** 10.3390/antibiotics14010071

**Published:** 2025-01-11

**Authors:** Alaaddin Korkut, Serap Özkaya Gül, Esra Aydemir, Hakan Er, Elif Odabaş Köse

**Affiliations:** 1 Department of Biology, Faculty of Science, Akdeniz University, 07058 Antalya, Turkey; 202451006003@ogr.akdeniz.edu.tr (A.K.); 202151006001@ogr.akdeniz.edu.tr (S.Ö.G.); esra@akdeniz.edu.tr (E.A.); 2 Department of Biophysics, Faculty of Medicine, Akdeniz University, 07070 Antalya, Turkey; hakaner@akdeniz.edu.tr; 3 Vocational School of Health Services, Akdeniz University, 07058 Antalya, Turkey

**Keywords:** *Acinetobacter baumannii*, antimicrobial resistance, colistin, *p*-coumaric acid, ferulic acid, *p*-methoxycinnamic acid

## Abstract

Colistin-resistant *Acinetobacter baumannii* (COLR-Ab) is an opportunistic pathogen commonly associated with nosocomial infections, and it is difficult to treat with current antibiotics. Therefore, new antimicrobial agents need to be developed for treatment. Based on this information, we investigated the antimicrobial, antibiofilm, and combination activities of *p*-coumaric acid (*p*-CA), ferulic acid (FA), and *p*-methoxycinnamic acid (*p*-MCA) against five COLR-Ab isolates. *p*-CA, FA, and *p*-MCA exhibited antimicrobial activity against COLR-Ab isolates, with minimum inhibitory concentration (MIC) values in the range of 256–128 µg/mL, 1024–512 µg/mL, and 512–128 µg/mL, respectively. The combination effects of the compounds with colistin (COL) were evaluated using a checkerboard synergy test. The combinations exhibited a synergistic effect and caused a 128- to 16-fold decrease in COL MIC values. In addition, the biofilm production capacities of the COLR-Ab isolates and the antibiofilm activities of the compounds were determined using the microtitre plate-based crystal violet (CV) technique. The compounds showed effective antibiofilm activity against strong and moderate biofilm-producing isolates, inhibiting biofilm formation by 77.5% and 19.7%. Spectrometric measurements were used to examine the effect of compounds on membrane permeability; 1.9-, 1.66-, and 1.34-fold increases in absorbance values were observed at MIC concentrations of *p*-CA, FA, and *p*-MCA, respectively. Furthermore, morphological changes caused by the compounds in the isolate were observed using scanning electron microscopy (SEM) micrographs. According to the WST assay, the compounds did not show any statistically significant cytotoxic effect on the cells (*p* > 0.05). These findings indicate that *p*-CA, FA, and *p*-MCA may be potential new alternative candidates against resistant *A. baumannii*.

## 1. Introduction

Infectious diseases have constituted a significant challenge to public health for centuries [1]. Although the development of antibiotics was thought to have effectively addressed this problem, the indiscriminate and overuse of antibiotics has led to concerns about antimicrobial resistance [2,3,4,5]. The rising prevalence of antibiotic resistance, particularly among clinically significant pathogens, such as multidrug resistant *Acinetobacter baumannii*, has prompted researchers to investigate the development of novel antimicrobial agents and/or approaches for the treatment of infectious diseases caused by these pathogens [6,7].

*A. baumannii* is a Gram-negative bacterium that causes nosocomial infections, including surgical site infections, bacteremia, ventilator-associated pneumonia, and urinary tract infections [8,9]. While *A. baumannii* was defined as a pathogen of minimal importance in the 1970s, in recent years, with the increasing use of antibiotics and invasive diagnosis and treatment methods, the number of multidrug resistant strains has been increasingly reported. This has led to an increase in both the frequency and severity of *A. baumannii* infections [10]. In addition, the ability of *A. baumannii* to form biofilms facilitates its persistence for prolonged periods in hospital settings, including medical devices and intensive care units. It is adept at surviving under highly dry conditions on abiotic surfaces [11]. The combination of *A. baumannii*’s biofilm-forming capabilities and its genetic plasticity provides this pathogen with an exceptional ability to develop resistance to the majority of antibiotics and disseminate these resistance patterns within hospital settings [12].

The carbapenem group of antibiotics represents one of the final treatment options available for the control of *A. baumannii* infections [13]. However, the rapid increase in carbapenem-resistant isolates detected in recent years has led the World Health Organization (WHO) to classify carbapenem-resistant *A. baumannii* as a “Priority 1 for new antibiotic research” group of “critical” pathogens in its 2017 report [14]. With the emergence of carbapenem-resistant Gram-negative pathogens, colistin (COL), which was previously discontinued in the 1980s due to its nephrotoxic and neurotoxic effects, has been reintroduced as a last resort for combating these pathogens [15].

COL is a cationic antimicrobial peptide that exhibits efficacy against the most Gram-negative bacteria [16,17]. Its polycationic characteristic enables it to disrupt the stabilization of LPS through electrostatic interactions with the negatively charged lipid A molecules in bacteria, thereby reducing the permeability of the outer membrane. COL then penetrates the inner membrane, resulting in the leakage of cell contents and subsequent cell lysis [18,19]. Nevertheless, the reuse of COL has resulted in the emergence of colistin-resistant organisms, with the number of strains increasing over the past decade [14]. Therefore, the development of novel antimicrobial agents and strategies to combat infections caused by colistin-resistant *A. baumannii* (COLR-Ab) is of paramount importance. In response to the increasing prevalence of antimicrobial resistance among persistent pathogens, the scientific community has turned to alternative approaches to combat antimicrobial resistance. In this context, a variety of alternative strategies have been proposed, including the use of medicinal plants with rich secondary metabolite content, target-specific antibodies, oligonucleotides and gene therapy systems, bacteriophages (modified phages and lysins), antimicrobial peptides, probiotics, and fecal transplantation therapy [20,21].

Secondary metabolites from plant extracts have an important place in the field of alternative antimicrobial strategies, largely due to their low toxicity and broad antimicrobial spectrum [22,23]. As a potential solution to the problem of antibiotic resistance, these bioactive substances, which increase antimicrobial activity, can be evaluated alone or in combination with antibiotics [24].

Cinnamic acids are aromatic carboxylic acids belonging to the phenolic acids class of plant secondary metabolites [25]. The compounds exhibit low polarity and water solubility, which can be attributed to the presence of a benzene ring and a short unsaturated hydrocarbon chain [26]. Cinnamic acids play a crucial role in plant physiology, including growth, development, reproduction, and the ability to tolerate environmental stress [27]. It has been demonstrated that cinnamic acid and its derivatives exhibit antimicrobial, antioxidant, anticancer, anti-inflammatory, and antidiabetic properties [25]. The antimicrobial and antibiofilm activity of *p*-coumaric acid (*p*-CA), ferulic acid (FA), and *p*-methoxycinnamic acid (*p*-MCA), which are among the cinnamic acids, has been demonstrated in numerous studies [28,29,30,31,32,33,34,35]. According to the literature, the mechanisms regarding the antimicrobial activities of cinnamic acids are increased cell membrane permeabilization, leakage of cytoplasmic components, and cellular morphological changes [35,36,37]. Molecular docking studies have demonstrated that these compounds also exhibit inhibitory effects on certain efflux pumps [38]. Moreover, *p*-CA has been shown to disrupt cellular processes by binding to the DNA double helix [35] and suppressing the expression of *RecA* [39]. In addition, the compounds contribute to antibiofilm activity by suppressing the expression of quorum sensing and biofilm-associated genes [40,41,42]. These compounds have also been demonstrated to possess antioxidant, neuroprotective, anticancer, and anti-diabetic effects [43,44,45].

The main focus of our study is to evaluate and compare the antimicrobial and antibiofilm activities of *p*-CA, FA, and *p*-MCA, which can be investigated as a potential treatment for COLR-Ab infections. The present study is the first to investigate the synergistic potential of *p*-CA, FA, and *p*-MCA in combination with COL. Furthermore, this study reveals whether the compounds increase the leakage of cell contents in the isolates through spectrophotometric measurements, and the morphological changes are shown using SEM observations. Consequently, this study also evaluates the potential of the compounds to damage cell membranes. Finally, the cytotoxic effects of the compounds on eukaryotic cells were investigated to demonstrate their safety potential.

## 2. Results

### 2.1. Antibacterial Susceptibility

The MIC values of COL, *p*-CA, FA, and, *p*-MCA against all bacterial strains are provided in Table 1. According to CLSI (2018) standards [46], the limit values of COL are as follows: ≤2 is considered sensitive, and ≥4 is considered resistant. The MIC value of COL for *Escherichia coli* NCTC 13846, which is used as a quality control strain, was determined as 4 μg/mL. COL exhibited antimicrobial efficacy against COLR-Ab isolates with MIC values ranging from 32 to 128 μg/mL. The COLR-Ab4 isolate showed the highest resistance to COL, while the COLR-Ab1 isolate exhibited the lowest resistance. The MIC value for *p*-CA was 256 μg/mL against all isolates except COLR-Ab5 (128 μg/mL). FA exhibited an MIC value of 1024 μg/mL against all isolates, except COLR-Ab4 isolate, with an MIC value of 512 μg/mL. The MIC values of *p*-MCA against all strains were 512 μg/mL for COLR-Ab1 and COLR-Ab2 isolates, 256 μg/mL for COLR-Ab3 and COLR-Ab4 isolates, and 128 μg/mL for the COLR-Ab3 isolate.

### 2.2. Evaluation of the Checkerboard Synergy Test Result

The activities of the combinations with COL of *p*-CA, FA, and *p*-MCA against five COLR-Ab isolates were evaluated using the checkerboard synergy test (Table 1). The results demonstrate that the combinations of COL + *p*-CA and COL + *p*-MCA exhibited synergistic activity against five strains. FICI values ranged from 0.16 to 0.28 for COL + *p*-CA and from 0.09 to 0.27 for COL + *p*-MCA. On the other hand, the combination of COL + FA showed a synergistic effect against four isolates with FICI values ranged from 0.12 to 0.15, while the effect against COLR-Ab4 was indifferent (FICI = 0.51).

### 2.3. Detection of Biofilm Production

**Figure 1:**
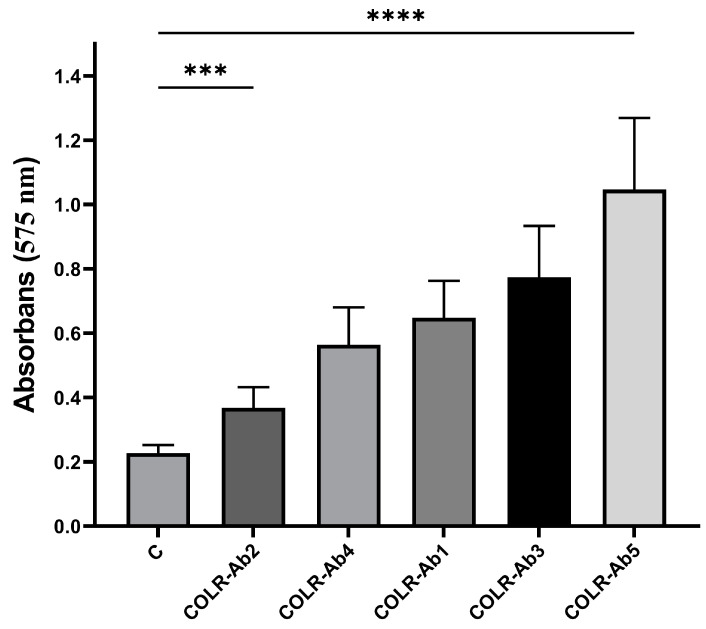
Biofilm formation ability of COLR-Ab isolates. *** *p* ≤ 0.001 and **** *p* ≤ 0.0001 were considered to indicate statistically significant differences compared to the control group.

The ability of COLR-Ab isolates to form biofilms was assessed using a microtiter plate-based crystal violet (CV) technique, with the findings compared to a negative control presented in Figure 1. According to the results, it was determined that COLR-Ab5 was a strong biofilm producer, COLR-Ab1, COLR-Ab3, and COLR-Ab4 were moderate biofilm producers, and COLR-Ab2 was a weak biofilm producer.

### 2.4. Antibiofilm Activity

**Figure 2:**
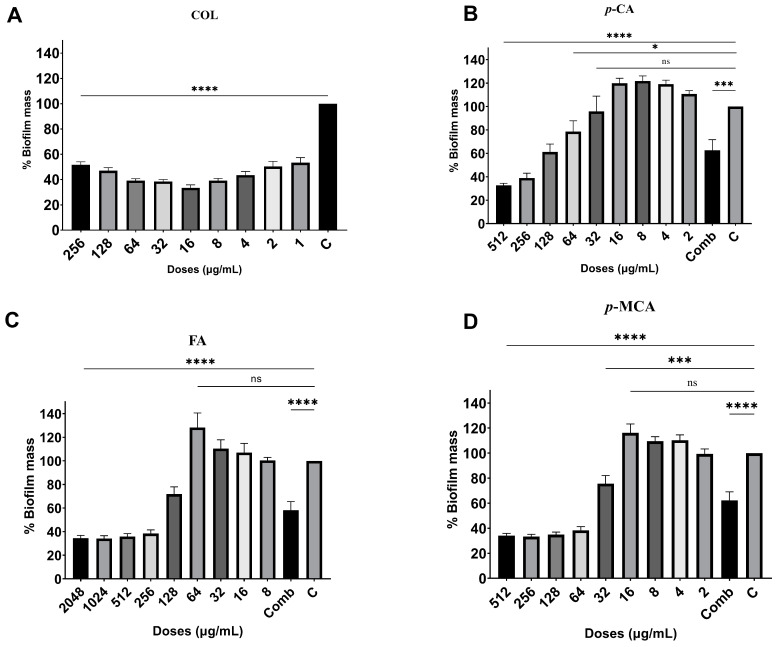
Antibiofilm activities of COL (**A**), *p*-CA (**B**), FA (**C**), and *p*-MCA (**D**) against COLR-Ab1 isolate. * *p* ≤ 0.05, *** *p* ≤ 0.001, and **** *p* ≤ 0.0001 were considered to indicate statistically significant differences compared to the growth control group. ns: not significant (*p* > 0.05); C, control; Comb, combination.

**Figure 3:**
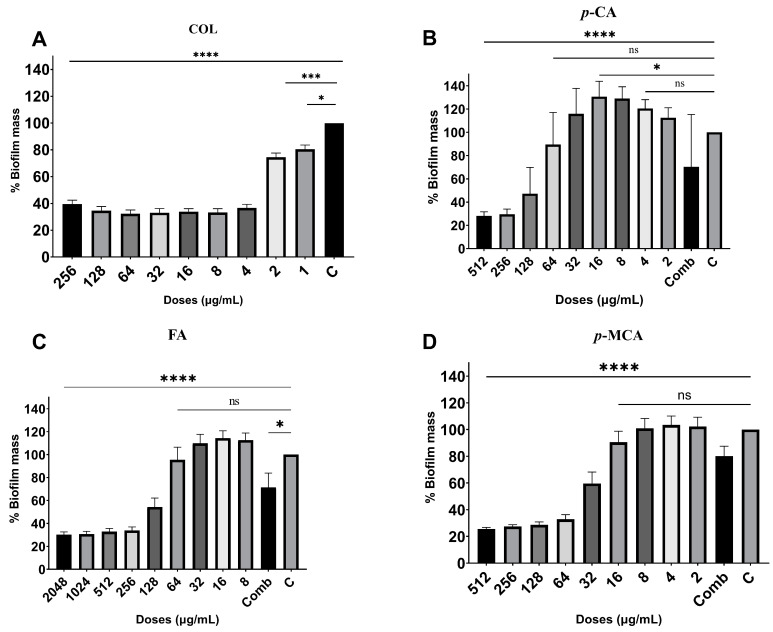
Antibiofilm activities of COL (**A**), *p*-CA (**B**), FA (**C**), and *p*-MCA (**D**) against COLR-Ab3 isolate. * *p* ≤ 0.05, *** *p* ≤ 0.001, and **** *p* ≤ 0.0001 were considered to indicate statistically significant differences compared to the growth control group. ns: not significant (*p* > 0.05); C, control; Comb, combination.

**Figure 4:**
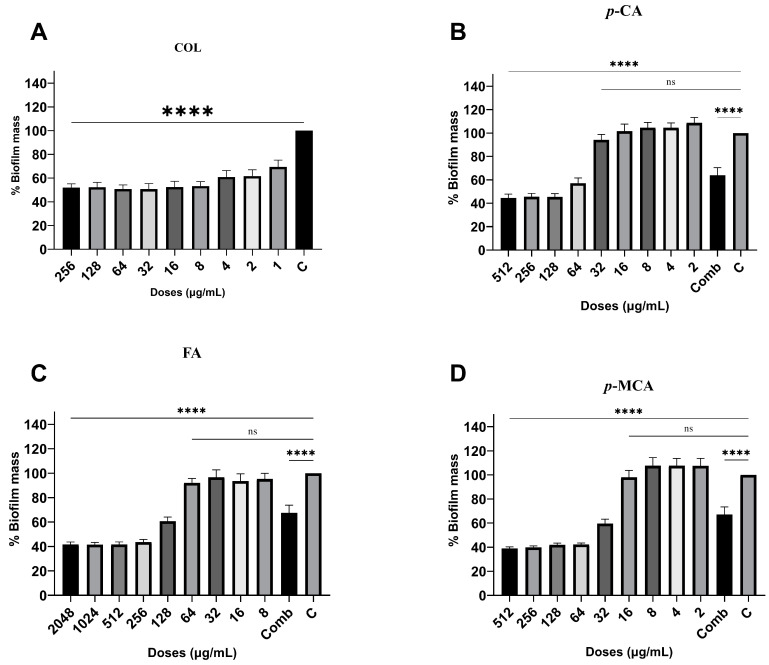
Antibiofilm activities of COL (**A**), *p*-CA (**B**), FA (**C**), and *p*-MCA (**D**) against COLR-Ab4 isolate. **** *p* ≤ 0.0001 were considered to indicate statistically significant differences compared to the growth control group. ns: not significant (*p* > 0.05); C, control; Comb, combination.

**Figure 5:**
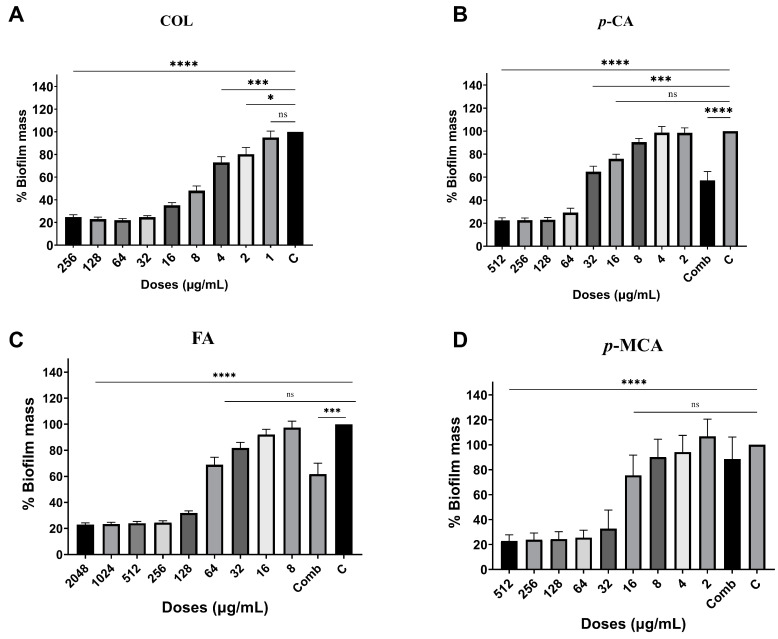
Antibiofilm activities of COL (**A**), *p*-CA (**B**), FA (**C**) and *p*-MCA (**D**) against COLR-Ab5 isolate. * *p* ≤ 0.05, *** *p* ≤ 0.001, and **** *p* ≤ 0.0001 were considered to indicate statistically significant differences compared to the growth control group. ns: not significant (*p* > 0.05); C, control; Comb, combination.

The antibiofilm activities of COL, *p*-CA, FA, and *p*-MCA against COLR-Ab isolates were determined by microtiter plate-based CV technique. The COLR-Ab2 isolate was not included in this study as it exhibited weak biofilm formation. The results show that all COL doses inhibited biofilm formation by approximately 19.7–75.2%, with the exception of the 1 μg/mL dose for COLR-Ab4 isolate. *p*-CA exerted a strong inhibitory effect by reducing biofilm formation of COLR-Ab isolates by 37.3–77.5% in the dose range of 512–32 μg/mL. However, at doses of 16–8 μg/mL of *p*-CA, a 20% increase in biofilm formation of COLR-Ab3 isolate was observed. FA showed significant antibiofilm activity in the dose range of 2048–128 μg/mL and inhibited biofilm formation by 28.1–77%. Finally, *p*-MCA was also able to reduce the biofilm formation of all isolates by 40.3–77.2% in the dose range 512–32 µg/mL. When the antibiofilm activities of the combinations were evaluated, it was determined that *p*-CA + COL inhibited biofilms by 29.7–42.7% in all isolates except COLR-Ab3 isolate. FA + COL successfully reduced biofilm formation at rates ranging from 28% to 41% for all isolates. *p*-MCA + COL exhibited effective biofilm inhibition only in COLR-Ab1 and COLR-Ab3 isolates with 41.7% and 32.8%, respectively. The antibiofilm activities of all agents against COLR-Ab isolates are presented in Figure 2, Figure 3, Figure 4 and Figure 5.

### 2.5. Measuring Cell Membrane Damage

**Figure 6:**
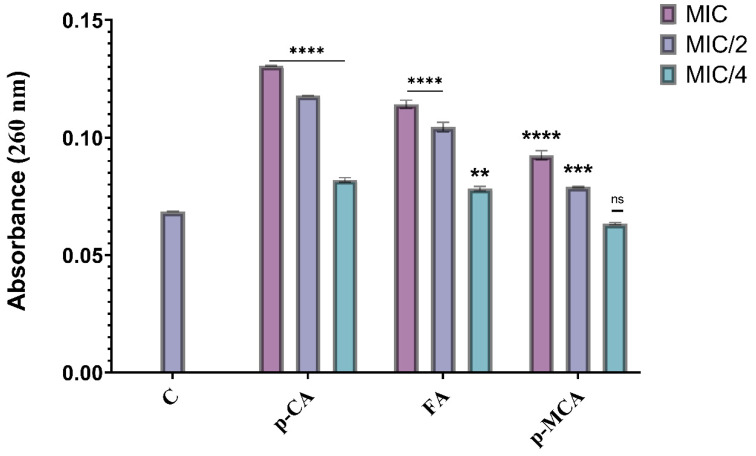
Absorbance values obtained by treating concentrations of *p*-CA, FA, and *p*-MCA at MIC, MIC/2, and MIC/4 values against the COLR-Ab4 isolate. ** *p* ≤ 0.01, *** *p* ≤ 0.001, and **** *p* ≤ 0.0001 were considered to indicate statistically significant differences compared to the control group. ns: not significant (*p* > 0.05); C, control.

In order to determine the damage caused by the agents (*p*-CA, FA, and *p*-MCA) on the cell membrane, the absorbance (amount of nucleic acid released from the cytoplasm) of the supernatant was measured at 260 nm. The results are presented in Figure 6, which shows the absorbance values obtained as a result of the application of MIC, MIC/2, and MIC/4 concentrations of all agents against the COLR-Ab4 isolate. Except for the absorbance value obtained with *p*-MCA application at the MIC/4 concentration, a significant difference was observed in all other absorbance values compared to the control (*p* > 0.05). The findings reveal that these agents caused a disruption to the membrane integrity of the COLR-Ab4 isolate, resulting in the release of intracellular components absorbed at 260 nm.

When COLR-Ab4 isolate was treated with MIC, MIC/2, and MIC/4 concentrations of *p*-CA, 1.9-, 1.71-, and 1.19-fold increase in absorbance values were observed, respectively (*p* ≤ 0.0001). The same three concentrations of FA (MIC, MIC/2, and MIC/4) caused 1.66-, 1.52-, and 1.14-fold increase in absorbance values, respectively. While 1.34- and 1.15-fold increases in absorbance values were showed at MIC and MIC/2 concentrations of *p*-MCA, respectively, no statistically significant differences were detected at MIC/4 concentrations of *p*-MCA compared to the control (*p* > 0.05).

### 2.6. Scanning Electron Microscopy

**Figure 7:**
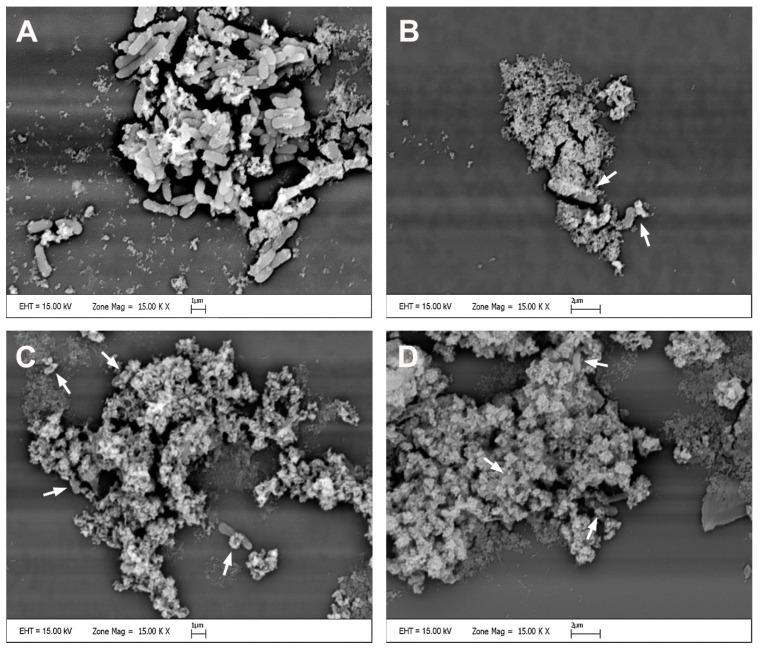
Scanning electron micrographs of the COLR-Ab4 isolate in the presence of MIC concentrations of *p*-CA, FA, and *p*-MCA: (**A**) untreated; (**B**) treated with *p*-CA; (**C**) treated with FA; (**D**) treated with *p*-MCA. White arrows indicate damaged bacterial cells. Scale bars, 1 µm in (**A**,**C**) and 2 µm in (**B**,**D**).

SEM analysis was performed to examine how the cell morphology of the COLR-Ab4 isolate changed when exposed to MICs of *p*-CA, FA and *p*-MCA. The results of SEM analysis are presented in Figure 7. The control group, untreated with any agent, had a rod-shaped cell morphology and cells that appeared smooth and regular (Figure 7A). Exposure to MIC concentrations of *p*-CA, FA, and *p*-MCA led to certain morphological changes in bacterial cells, compared to untreated control bacteria. After treatment with these agents, the general appearance of the remaining cells showed shrinkage, collapses, gaps, and detachments due to disruption of the cell wall, degeneration of the cell membrane, and a decrease in the amount of cytoplasm (Figure 7B–D).

### 2.7. Results of Cytotoxic Activity

**Figure 8:**
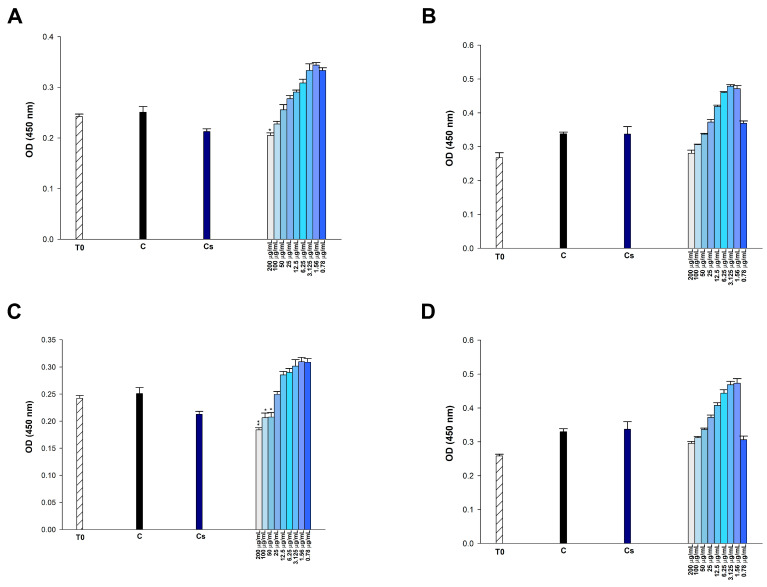
Effect of (**A**) etoposide, (**B**) *p*-CA, (**C**) FA, and (**D**) *p*-MCA on cell viability in 293T cells after 24 h of incubation. * *p* < 0.05 and ** *p* < 0.01 were considered to indicate statistically significant differences compared to the control group.

**Figure 9:**
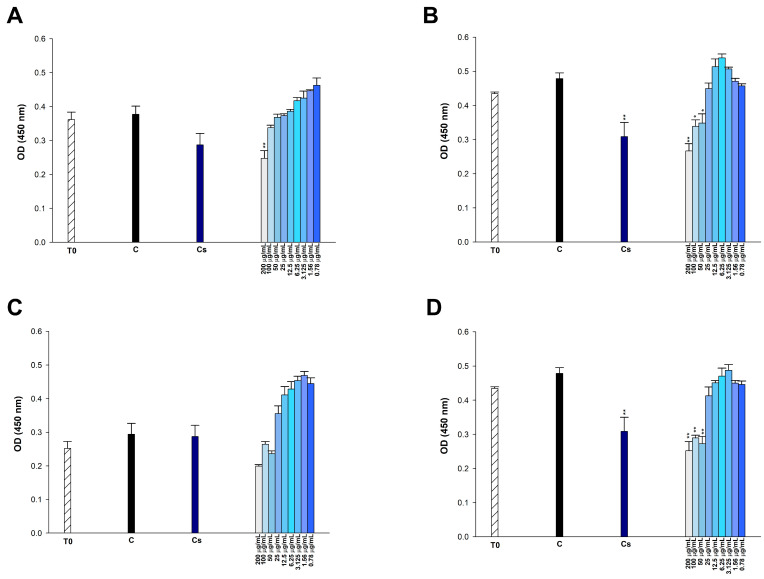
Effect of (**A**) etoposide, (**B**) *p*-CA, (**C**) FA, and (**D**) *p*-MCA on cell viability in HUVEC cells after 24 h of incubation. * *p* < 0.05 and ** *p* < 0.01 were considered to indicate statistically significant differences compared to the control group.

**Figure 10:**
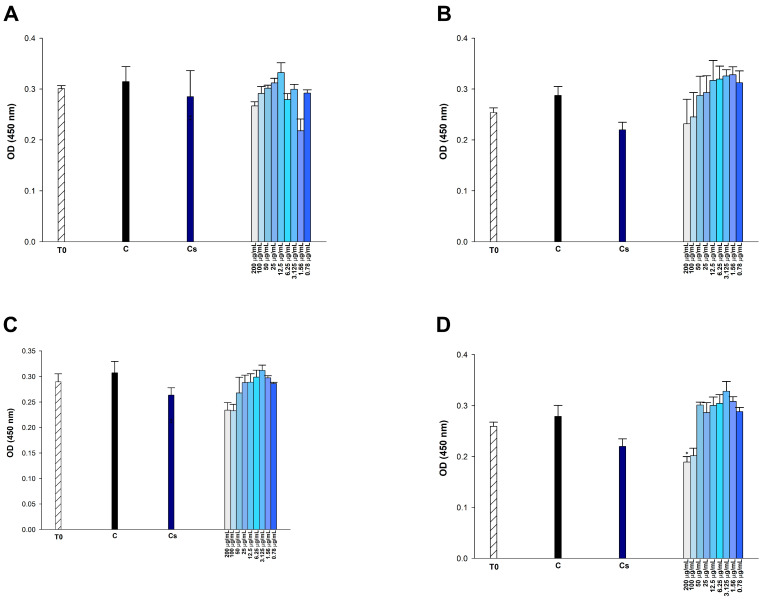
Effect of (**A**) etoposide, (**B**) *p*-CA, (**C**) FA, and (**D**) *p*-MCA on cell viability in Vero cells after 24 h of incubation. * *p* < 0.05 was considered to indicate statistically significant differences compared to the control group.

To examine the cytotoxic potential of *p*-CA, FA, *p*-MCA, and etoposide, we incubated the cells with varying concentrations of compounds (0.78125–200 μg/mL) for 24 h and then performed the CCK-8 assay, which indirectly indicates metabolically active cells in vitro. In 293T cells, none of the administered doses of *p*-CA and *p*-MCA induced cytotoxicity, but FA exhibited cytotoxic effects at doses of 200 μg/mL (*p* < 0.01), 100 μg/mL (*p* < 0.05), and 50 μg/mL (*p* < 0.05) (Figure 8). FA did not induce cytotoxic effects on HUVEC cells at any tested doses. *p*-MCA induced cytotoxicity in these cells at concentrations of 200, 100, and 50 μg/mL (*p* < 0.01). Similarly, *p*-CA showed cytotoxicity in HUVEC cells at concentrations of 200 μg/mL (*p* < 0.01), 100 μg/mL (*p* < 0.05), and 50 μg/mL (*p* < 0.05) (Figure 9). FA and *p*-CA exhibited no cytotoxic effects on Vero cells at any administered concentrations, whereas only 200 μg/mL *p*-MCA induced cytotoxicity in Vero cells (Figure 10).

## 3. Discussion

Over the past two decades, the prevalence of antimicrobial resistance has increased as a consequence of the unnecessary overuse of antibiotics. The phenomenon has spread rapidly among pathogens, leading to a rise in morbidity and mortality from infectious diseases [47]. By 2050, it is estimated that 10 million deaths will occur annually worldwide due to antibiotic resistance, which has become one of the most urgent global concerns of the 21st century [48,49]. In addition, while antimicrobial resistance is gradually increasing, the rate of discovery of new antibiotics has gradually decreased [50]. Secondary metabolites obtained from plants represent a promising new source of antimicrobial agents, exhibiting high antimicrobial activity and low toxicity [23]. The aim of our study is to investigate the antimicrobial and antibiofilm activities of various cinnamic acid derivatives (*p*-CA, FA, and *p*-MCA) against COLR-Ab isolates, to reveal their combination activities with COL, and to evaluate the potential of these compounds to damage the bacterial membrane. Broth microdilution test was conducted to ascertain the COL resistance levels of *A. baumannii* isolated from hospital samples. MIC values for COL were determined for all samples, with values ranging from 32 to 128 µg/mL. In accordance with the Clinical & Laboratory Standards Institute (CLSI) standards, all *A. baumannii* isolates studied exhibited COL resistance [46]. Antimicrobial activity was observed against all *A. baumannii* strains with MIC values in the range of 256–128 µg/mL for *p*-CA, 1024–512 µg/mL for FA, and 512–128 µg/mL for *p*-MCA. In the literature, Ibitoye and Ajiboye (2019) found that FA was effective against various *A. baumannii* strains with MIC values between 256 and 64 µg/mL [28]. In another study, the MIC value of FA was determined to be 12.5 μg/mL against three *A. baumannii* isolates. This value is considerably lower than the MIC results obtained in the present study [51].

It has been determined by many researchers that *p*-CA, FA, and *p*-MCA have antimicrobial activity against various bacteria [29,30,32,33,34,52]. Lou et al. (2012) determined the MIC values of *p*-CA as 80 μg/mL, 10 μg/mL, and 20 μg/mL for Gram-positive bacteria *S. aureus*, *Streptococcus pneumoniae*, and *Bacillus subtilis*, and 20 μg/mL for Gram-negative bacteria *E. coli*, *Shigella dysenteriae*, and *Salmonella typhimurium*, respectively [35]. In a study conducted by Nakazono et al. (2006), the MIC values of *p*-MCA against *Micrococcus luteus*, *S. aureus*, *E. coli*, and *Salmonella enteritidis* were determined as 80 μg/mL, 60 μg/mL, 50 μg/mL and 60 μg/mL, respectively [29]. In another study with *p*-MCA, MIC values were determined as 30 μg/mL against *E. coli* and 40 μg/mL against *S. aureus* and *B. subtilis* [30]. In contrast, these studies demonstrate that the compounds are more sensitive to other bacteria than *A. baumannii*.

The structure–function relationships of various phenolic acids are thought to affect antibacterial activity. The results of the study conducted for this purpose by Sanchez-Maldonado et al. (2011) indicate that the activity of hydroxycinnamic acids was not significantly affected by reducing the number of hydroxyl groups. Furthermore, substitution of hydroxyl groups with methoxy groups did not result in a significant increase in activity [34]. In our study, we can classify the antimicrobial activities of cinnamic acids as *p*-CA > *p*-MCA > FA. Considering these results, it can be said that hydroxy (*p*-CA) and methoxy (*p*-MCA) substitutions in the aromatic ring in the main structure of cinnamic acids do not significantly affect the antimicrobial activity.

Today, it is known that antibiotic-based combination therapies are applied as a suitable option in the treatment of multidrug-resistant *A. baumannii* infections to increase the success of clinical treatment. Based on this, combination studies of a natural compound and an antibiotic have become a widely researched topic. The synergistic effect between plant-derived compounds and antibiotics may lead to the reuse of an antibiotic that is inadequate for treatment alone. According to the results of our study, in which we investigated the combinations of three compounds with COL for this purpose, all other combinations showed synergistic effect except for the combination of COL and FA on COLR-Ab4 isolate. The combination of COL and FA exhibited an indifferent effect only against this isolate (FICI = 0.51). The combination of the three compounds with COL resulted in a remarkable reduction in the MIC of this antibiotic. It was observed that COL MIC values in the range of 128–32 µg/mL decreased to 4–1 µg/mL. COL MIC values decreased 16–64 fold in COL and *p*-CA combinations, 16–128 fold in COL and FA combinations, and 32–64 fold in COL and *p*-MCA combinations. We think these results are very important, because COL is an antibiotic that was introduced several decades ago and subsequently withdrawn from the market due to reports of significant nephrotoxicity and neurotoxicity [53,54]. The majority of infections caused by multidrug-resistant and pandrug-resistant *A. baumannii* are reported in critical care unit patients [10]. Long-term use of COL to treat these infections has been associated with the development of nephrotoxic and neurotoxic side effects [55]. Consequently, the significant reduction in the MIC values of COL in combination with *p*-CA, FA, and *p*-MCA represents a promising result in terms of the safe use of this antibiotic in treatment.

No other studies investigating the combined activity of COL with these compounds against COLR-Ab clinical strains were found in the literature. In this context, our study is the first study to investigate the combination activities of *p*-CA, FA, and *p*-MCA with COL against *A. baumannii*. In the study by Ibitoye and Ajiboye (2019), the combination of quinolone-based antibiotics with FA exhibited synergistic effect against various *A. baumannii* isolates [28]. There are studies observing the synergistic interactions of different phenolic compounds with COL against *A. baumannii* isolates. Synergistic activity has been shown in the combination of some phenolic acids such as caffeic acid, gallic acid, and protocatechuic acid with COL [56]. Köse et al. (2023) found that the combination of COL and quercetin exhibited a synergistic effect against COLR-Ab isolates and a 4- to 16-fold decrease in COL MIC values [57]. In another study, the combination of COL with cinnamic acid against MDR *A. baumannii* isolates was determined to exhibit a 4- to 32-fold reduction in COL MIC values in five of the isolates [42].

Biofilm formation ability of *A. baumannii* is an essential factor contributing to the survival of these bacteria on abiotic surfaces in hospital environments under very dry conditions, their easy spread in these environments, and their antibiotic resistances [58,59]. Studies reveal that *A. baumannii* strains have a high biofilm formation capacity. Bala et al. (2016) found that 40% of 75 clinical isolates were strong, 12% were moderate, 16% were weak biofilm producers, and only 32% did not produce any biofilm. MDR was observed in 63% of all isolates [60]. Another study reported that 58% of 100 clinical isolates of *A. baumannii* collected from patients in intensive care units were capable of forming biofilms, and all of these isolates had widespread antibiotic resistance [61]. In a study by Qi et al. (2016), biofilm formation was detected in 249 out of 272 *A. baumannii* clinical isolates collected from various hospitals in China [12]. As a result of our study, it was determined that one of the five isolates was a strong, three were medium, and one was a weak biofilm producer.

In the next step of our study, we also investigated the antibiofilm activity of the agents on the strains that we identified as strong and medium biofilm strains. The results show that COL significantly and dose-dependently reduced biofilm formation. In contrast, several studies in the literature reported that COL alone did not significantly reduce biofilm formation of *A. baumannii* and even showed higher inhibition at lower COL doses [62,63]. The tested *p*-CA, FA, and *p*-MCA also considerably inhibited biofilm formation of COLR-Ab isolates in a dose-dependent manner. Mumtaz et al. (2023) also found that FA inhibited biofilm production of *A. baumannii* isolates by 49–69% [51]. Combinations of COL with the compounds we tested also exhibited a remarkable biofilm inhibition. The ability of microorganisms such as *A. baumannii* to form biofilms is a contributing factor to antibacterial resistance and treatment failures. Therefore, the discovery of effective biofilm-inhibiting agents may provide potential benefits both in the treatment of biofilm-associated infections and in slowing the rate of spread of antibiotic resistance. Based on our results, we can say that *p*-CA, FA, and *p*-MCA exhibiting biofilm inhibition at MIC, FIC and combination concentrations are potential antibiofilm agents. Although the studies with *A. baumannii* are quite limited in the literature, the antibiofilm activities of these compounds have been studied against many pathogens. In these studies, *p*-MCA on *Chromobacterium violaceum* [64] and *p*-CA and FA on *E. coli* [65] and *Salmonella enteritidis* [66] were found to cause biofilm inhibition.

Due to their hydrophobic character, cinnamic acids cause bacterial death through the leakage of intracellular components by forming local pores or ruptures in the cell membrane. A number of studies have shown that cells exposed to *p*-CA and FA undergo irreversible destruction by reducing the negative charge of the cell surface [36], inducing a significant increase in plasma membrane permeability [35] and causing changes in cell morphology [37]. Moreover, Hemaiswarya and Doble (2010) showed that the combinations of *p*-CA and FA with various antibiotics caused damage to bacterial membranes and the hydrophobic nature of the compounds increased this damage [67]. In the present study, in order to determine whether the tested compounds caused damage to the bacterial cell membrane, the absorbance (amount of nucleic acid released from the cytoplasm) of the supernatant obtained after treatment of the bacteria with the compounds was measured at 260 nm. Our results show that the compounds cause membrane damage in *A. baumannii*. When COLR-Ab4 isolate was treated with the compounds, a significant increase in absorbance values was detected compared to the control (except MIC/4 concentration of *p*-MCA). That is, these compounds disrupted the membrane integrity of the COLR-Ab4 isolate, causing the release of intracellular components from the cell. Images obtained using SEM also showed several morphological changes in bacterial cells exposed to the compounds compared to untreated control bacteria. After the application of the compounds, collapses, wrinkles, gaps, and ruptures were observed in the appearance of bacterial cells. Therefore, the antimicrobial effects of *p*-CA, FA, and *p*-MCA are thought to be related to bacterial membrane damage, increased membrane permeability, and leakage of cellular contents from the cell, resulting in bacterial death.

This study assessed whether these compounds (*p*-CA, FA, and *p*-MCA), demonstrated to possess antibacterial activities against bacteria, exhibit cytotoxic effects on three normal eukaryotic cells. *p*-CA did not exhibit cytotoxic effects on 293T and Vero cells at any dose within the tested range (0.78–200 μg/mL) following a 24 h incubation period (*p* > 0.05). Therefore, the IC_50_ value for *p*-CA was indeterminate in 293T and Vero cells. It was reported that 200 and 150 μg/mL doses of *p*-CA were cytotoxic in HK-2 kidney epithelial cells [68]. However, herein, we demonstrate that *p*-CA did not induce cytotoxic effects in 293T cells at any tested doses. This discrepancy may stem from the fact that the kidney cells utilized by the researchers were derived from an adult human, but the 293T cells employed in our study were embryonic. In accordance with our results, Saenglee et al. reported that the IC_50_ values of *p*-CA were greater than 5 mM, the maximum tested dose, in Vero cells after 24 h of incubation [69]. Sevimli-Gur and Yesil-Celiktas reported the IC_50_ value for the cytotoxic effects of *p*-CA in Vero cells after 48 h as 163 ± 0.3 μg/mL [70]. However, *p*-CA was found to be cytotoxic at 200 (*p* < 0.01), 100 (*p* < 0.05), and 50 (*p* < 0.05) μg/mL in HUVEC cells. Posadino et al. also reported that *p*-CA was cytotoxic in HUVEC cells at doses of 10 and 25 mM, while no statistically significant cytotoxicity was observed at lower doses [71]. Kong et al. investigated the cytotoxicity of the *p*-CA on the growth of HUVEC-originated ECV304 cells. They declared that *p*-coumaric acid had no significant effects on the cell viability up to concentrations of 2.5 mM, whereas a high concentration of *p*-CA (5 mM) was shown to induce cytotoxicity in ECV304 cells [72]. FA, after 24 h of incubation, did not exhibit any cytotoxic effect on Vero and HUVEC cells at any dose (*p* > 0.05), while it was found to be cytotoxic in 293T cells at concentrations of 200 (*p* < 0.01), 100 (*p* < 0.05), and 50 (*p* < 0.05) μg/mL. Similarly, FA exhibited cytotoxicity in HK-2 cells, human kidney epithelial cells, at concentrations of 200, 150, and 75 μg/mL [68]. It has been reported that the IC_50_ value of FA in Vero cells is above 5 mM, the maximum tested dose, after 24 h of incubation [69]. The IC_50_ value for the cytotoxic effects of FA in Vero cells after 48 h was found to be 134 ± 0.8 μg/ml [70]. *p*-MCA, after 24 h, showed no cytotoxic effect at any dose tested in 293T cells (*p* > 0.05). *p*-MCA exhibited cytotoxic effects in HUVEC cells at doses of 200, 100, and 50 μg/mL (*p* < 0.01), and only at 200 μg/mL (*p* < 0.05) in Vero cells after 24 h.

## 4. Materials and Methods

### 4.1. Bacterial Strains and Test Compounds

Five COLR-Ab strains isolated from various clinical specimens were obtained between 2016 and 2017 from the Microbiology Department of the Central Laboratory of Akdeniz University Hospital, Antalya, Turkey. All isolates were isolated from tracheal samples obtained from patients hospitalized in the intensive care unit. The stock solutions of *A. baumannii* strains were cultivated in blood agar (Becton Dickinson, Franklin Lakes, NJ, USA) at 35 ± 2 °C for 18–24 h. Identification of the colonies was confirmed using Matrix-Assisted Laser Desorption/Ionization Time-of-Flight (MALDI-TOF) mass spectroscopy (Bruker Daltonics, Bremen, Germany). A BD Phoenix100 automated system (Becton Dickinson, Franklin Lakes, NJ, USA) was used to determine the susceptibilities of bacteria for seven antibiotics, namely amikacin, ciprofloxacin, gentamicin, imipenem, meropenem, netilmicin, and trimethoprim/sulfamethoxazole. The automated system was evaluated according to the criteria of the CLSI. The colistin broth disk elution method was used to test COL sensitivity, and all five isolates selected for the study were resistant to COL. The antimicrobial resistance profiles of the isolates are given in Table 2. *E. coli* NCTC 13846 COL was used as a quality control strain in broth dilution method to verify the MIC values. The isolates were stored at −80 °C until use and sub-cultured on blood agar for in vitro testing.

COL was obtained from Sigma-Aldrich (St. Louis, MO, USA), while *p*-CA, FA, and *p*-MSA were obtained from BostonChem (Boston, MA, USA). Stock solutions of 512 µg/mL for COL were prepared and stored at −20 °C. Stock solutions of 1024 µg/mL for *p*-CA and *p*-MCA and 4096 µg/mL for FA were prepared using pure ethanol and stored at −20 °C.

### 4.2. MIC Determination

The broth microdilution method, as recommended by the CLSI, was used to determine the MIC values of COL, *p*-CA, FA, and *p*-MCA [46]. The serial two-fold dilutions of antimicrobial agents were prepared using 96-well microplates with cation-adjusted Mueller-Hinton Broth (MHB) (CAMHB, Merck KGaA, Darmstadt, Germany). Firstly, an initial concentration of 8192 µg/mL was chosen to determine the MIC values of all compounds against COLR-Ab isolates. Subsequent to the initial MIC results, concentration ranges including the MIC value of each compound were determined as 256–0.5 µg/mL for COL, 2048–4 µg/mL for FA, and 512–1 µg/mL for *p*-CA and *p*-MCA. The bacterial suspension was adjusted to the 0.5 McFarland standard and added to each well (final bacterial concentration: 5 × 10^5^ colony forming units [CFU]/mL). Additionally, the microdilution plates included controls for bacterial growth (CAMHB + bacteria) and medium sterility (CAMHB). Microdilution plates were incubated at 35 ± 2 °C for 18–24 h. The MIC values were calculated by comparing the growth densities in the antibiotic-containing wells to those in the control wells used in each test set. Each experiment was conducted in triplicate.

### 4.3. Checkerboard Synergy Test

The checkerboard synergy test, based on microdilution, was carried out to investigate the combined activity of antibiotic and cinnamic acid derivatives. The effectiveness of the two antimicrobial drugs in combination was evaluated using a 96-well microplate for each strain. The medium employed was CAMHB. The combination of the two agents was examined within the dilution range of 2 × MIC and 0.0625 × MIC. COL was added vertically, and cinnamic acids was added horizontally to the wells. The bacterial suspension was prepared to 0.5 McFarland standard density, and the final bacterial concentration was adjusted to 5 × 10^5^ CFU/mL. In total, 10 µL of bacterial inoculum was added to each well. Bacterial growth control (CAMHB + bacteria) and medium sterility control (CAMHB) were also examined for each plate. The plates were incubated at 35 ± 2 °C for 18–24 h. Each experiment was conducted in triplicate.

The fractional inhibition concentrations (FIC) of all antimicrobial agents were calculated for the evaluation of the results in accordance with the following formulae:

FIC_A_ = (MIC of A in combination/MIC of A alone)

FIC_B_ = (MIC of B in combination/MIC of B alone)

FIC_index_(FICI) = FIC_A_ + FIC_B_

FICI ≤ 0.5 was considered to indicate synergism, 0.5 ≤ FICI ≤ 4 was considered to indicate indifference, and FICI > 4 was considered to indicate antagonism [73]. In the formulae, the letter “A” is used to represent COL, while “B” is used to represent one of the compounds, *p*-CA, FA, and *p*-MCA.

### 4.4. Detection of Biofilm Production

To determine the biofilm formation ability of COLR-Ab isolates, a 96-well microtiter plate-based CV assay was applied with minor modifications to the method described by Kırmusaoğlu (2019) [74]. Briefly, bacteria were inoculated into MHB medium containing 0.2% glucose and incubated at 37 °C for 16–20 h. The bacterial suspension, adjusted to 0.5 McFarland using the same medium, was prepared to a final concentration of 5 × 10^5^ CFU/mL and inoculated into 96-well flat-bottom microtiter plates. Wells containing only medium (MHB) were used as negative control. The plates were then incubated overnight at 35 ± 2 °C in a normal atmosphere.

Following the incubation period, the microtiter plates were gently washed three times with PBS. Subsequently, 200 µL of methanol (99%) was added to each well and left for 15 min. The methanol was then removed from the wells and the plate was left to dry for approximately 30 min, thus completing the fixation process. Then, 200 µL of 0.1% CV solution was added to each well and incubated for 20 min at room temperature. The plates were washed three times with PBS in order to remove the excess stain, and then 150 μL of 95% ethanol was added to the wells and incubated for 20 min. The optical density of the CV-stained biofilm was measured at a wavelength of 575 nm using a Thermo Labsystem Multiscan Spectrum microtiter plate reader (Thermolabsystem, Chantilly, VA, USA). Each experiment was conducted in triplicate.

### 4.5. Antibiofilm Activity

In order to evaluate the antibiofilm activity of COL, *p*-CA, FA, and *p*-MCA, a 96-well microtiter plate-based CV test was carried out, with minor modifications to the methods previously described by Kırmusaoglu (2019) and Haney et al. (2021) [74,75]. Briefly, bacteria were inoculated into MHB medium containing 0.2% glucose and incubated at 37 °C for 16–20 h. The bacterial suspension, adjusted to 0.5 McFarland using the same medium, was prepared to a final concentration of 5 × 10^5^ CFU/mL and inoculated into 96-well flat-bottom microtitre plates. The antibacterial activity of the agents was examined at a dilution range of 2 × MIC and 0.0625 × MIC. Wells containing only medium were used as negative controls (MHB), and wells containing only medium and bacteria were used as growth controls. The plates were then incubated overnight at 35 ± 2 °C in a normal atmosphere. Following incubation, CV staining procedures were carried out in a similar manner to Section 4.4.

The antibiofilm index was calculated using the following formula:

% Biofilm Mass = (100 × Sample OD_575_/Growth Control OD_575_)

### 4.6. Cytoplasmic Membrane Permeability Assay

A cell membrane permeability assay was carried out in accordance with the methods described by Köse (2023) [57]. Membrane damage measurements were performed on COLR-Ab4, which was found to be the most resistant strain to COL. The bacteria were firstly incubated in MHB at 35 ± 2 °C overnight. Subsequently, the bacterial culture was centrifuged at 4000× *g* at 15 min, after which the pellet was washed twice with PBS. The bacterial suspensions were treated with the determined concentrations of agents, specifically 1 × MIC, 1/2 × MIC and 1/4 × MIC. A suspension containing PBS and bacteria was used as a control. All samples were incubated at 35 ± 2 °C for 3 h, centrifuged at 13,400× *g* for 15 min, and the supernatant was harvested. The absorbance (A)_260_ of the supernatant was quantified using a UV-Vis spectrophotometer (Agilent Technologies, Santa Clara, CA, USA) to determine the amount of nucleic acid released from the cytoplasm. Each experiment was repeated three times.

### 4.7. Scanning Electron Microscopy

To examine the potential impact of the antimicrobial agents on the cell morphology of the COLR-Ab4 isolate, SEM studies were carried out according to the method Bendali et al. (2008), with minor modifications [76]. In this assay, the bacteria were incubated for 3 h at 35 ± 2 °C in MHB at MIC values (256 µg/mL for *p*-CA and *p*-MCA, 512 µg/mL for FA). Bacteria incubated without treatment with agents were used as controls. After incubation, the suspension was centrifuged for 10 min at 4000× *g*. The bacteria were washed twice with 0.1 M phosphate-buffered solution (PBS, pH 7.0). The pellet was fixed in 2.5% glutaraldehyde solution and kept for at 4 °C for 2 h. After washing the bacteria again twice with PBS, the sample was fixed in 1% osmium tetroxide solution for 1 h. Then, the samples were dehydrated through suspension in increasing concentrations of ethanol (30, 50, 70, 80, 90, and 100%) for 10 min each. The ethanol was then replaced by 100% acetone. Finally, the samples were fixed on an SEM support, coated with gold/palladium using sputtering in a vacuum, and examined using SEM (Zeiss LEO 1430, Cambridge, UK).

### 4.8. Cell Culture

293T [HEK-293] (ATCC^®^ CRL-1573), HUVEC (ATCC CRL-1730 ™, human umbilical vein endothelial cells) and Vero (ATCC^®^ CCL-81™, African green monkey kidney epithelial cells) cells were used in this study. Cells were cultured in RPMI-1640 medium supplemented with 10% fetal bovine serum, 0.1 mM non-essential amino acids, 5% sodium pyruvate, 2 mM L-glutamine, and 10 μg/mL gentamicin in a humidified atmosphere of 5% CO_2_, 95% air at 37 °C. After growing to approximately 80% confluence, the cells were detached with 0.25% (*w*/*v*) trypsin-EDTA and then subcultured. The cells were stored in a deep freezer at −80 °C in a solution prepared with 95% medium and 5% DMSO.

### 4.9. Cell Viability Assay

Cell viability was assessed according to the manufacturer’s protocol using a Cell Counting Kit-8 (CCK-8) (KTA1020, Abbkine, Atlanta, GA, USA). The assay is based on the reduction of 2-(2-methoxy-4-nitrophenyl)-3-(4-nitrophenyl)-5-(2,4-disulfophenyl)-2H-tetrazolium monosodium salt (WST-8) by cellular mitochondrial dehydrogenase [77]. Cells were seeded in 96-well plates at a final concentration of 5 × 10^3^ cells/well in 100 μL complete culture medium and were allowed to attach for 24 h. After the cells reaching to 80–90% confluence, the medium was removed and the cells were treated with *p*-CA, FA, *p*-MCA, and etoposide (0.78125, 1.5625, 3.125, 6.25, 12.5, 25, 50, 100, and 200 μg/mL) prepared in 1% FBS containing complete medium for 24 h. Immediately after the treatment, cell viability in a single column was determined and recorded as time zero (T_0_). The plain medium with 1% FBS was used as a negative control, whereas ethanol was used as solvent control. Each treatment was performed in four well replicates. At the end of the incubation, the medium was gently aspirated to terminate the experiment, a total volume of 10 μL (5 mg/mL) of CCK-8 solution was added to each well and further incubated under the same conditions for 4 h. The absorbances at 450 nm were measured in a microplate reader (Thermo Labsystem, Waltham, MA, USA), using wells without cells as background. The sample readings were calculated by subtracting the average of background absorbances. The half-maximal inhibitory concentration (IC_50_) of each extract was derived by a nonlinear regression model (curve-fit) based on a sigmoidal dose response curve (variable slope) and was computed using Graph-Pad Prism, version 4.00 (Graph-Pad Software, San Diego, CA, USA). Cell viability was calculated using the following formula: Cell viability (%) = (OD_treatment_ − OD_blank_)/(OD_control_ − OD_blank_) × 100% [78].

### 4.10. Statistical Analyses

All measurements were expressed as their mean ± SEM. Data were analyzed using one-way ANOVA followed by Dunnett’s Multiple Comparisons Test for comparisons of group means to control (Graph Pad InStat., San Diego, CA, USA). A *p*-value of <0.05 was considered statistically significant. Best-fit graphs were generated using SigmaPlot 10.0 (Systat Software, San Jose, CA, USA) and GraphPad Prism 9.0 (GraphPad Software, Inc., San Diego, CA, USA).

## 5. Conclusions

The results reported in this study indicate that *p*-CA, FA, and *p*-MCA show antimicrobial and antibiofilm effects on COLR-Ab isolates. The combinations of these compounds with COL demonstrated a synergistic effect against the resistant strains tested and succeeded in reducing the MIC values of COL to susceptible ranges. In light of the increasing phenomenon of resistance to existing antibiotics, this study suggests that these compounds show promise for the development of new antimicrobial agents for the treatment of COLR-Ab infections. Further studies are necessary for the optimization of these compounds and the evaluation of their efficacy and safety using in vivo models.

## Figures and Tables

**Table 1:** Results of antibacterial activities of COL, *p*-CA, FA, and *p*-MCA and their combinations against COLR-Ab strains.

Strains	MIC (µg/mL)				Mean FICI for COL Combined with:		
	COL	p-CA	FA	p-MCA	p-CA	FA	p-MCA
COLR-Ab1	32	256	1024	512	0.16 (S)	0.15 (S)	0.09 (S)
COLR-Ab2	64	256	1024	512	0.19 (S)	0.15 (S)	0.09 (S)
COLR-Ab3	64	256	1024	256	0.16 (S)	0.15 (S)	0.27 (S)
COLR-Ab4	128	256	512	256	0.15 (S)	0.51 (I)	0.14 (S)
COLR-Ab5	64	128	1024	128	0.28 (S)	0.12 (S)	0.27 (S)
*E. coli*	4	-	-	-	-	-	-

S: synergistic effect; I: indifferent effect.

**Table 2:** The antimicrobial resistance profiles for COLR-Ab isolates.

Isolates	MIC (µg/mL)							
	AMK	CIP	GEN	IPM	MEM	NET	SXT	COL *
COLR-Ab1	≤4 (S)	≤0.125 (S)	≤1 (S)	≤0.25 (S)	≤0.125 (S)	1 (S)	≤1/19 (S)	>4 (R)
COLR-Ab2	>16 (R)	>2 (R)	>4 (R)	>8 (R)	>8 (R)	>4 (R)	>4/76 (R)	>4 (R)
COLR-Ab3	>16 (R)	>2 (R)	>4 (R)	4 (I)	>8 (R)	>4 (R)	>4/76 (R)	>4 (R)
COLR-Ab4	>16 (R)	>2 (R)	>4 (R)	>8 (R)	>8 (R)	>4 (R)	>4/76 (R)	>4 (R)
COLR-Ab5	>16 (R)	>2 (R)	>4 (R)	>8 (R)	>8 (R)	>4 (R)	>4/76 (R)	>4 (R)

AMK, Amikacin; CIP, Ciprofloxacin; GEN, Gentamicin; IPM, Imipenem; MEM, Meropenem; NET, Netilmicin; SXT, Trimethoprim/sulfamethoxazole; COL, Colistin; R, Resistant; I, Intermediate; S, Susceptible; * Colistin broth disk elution method was applied to test susceptibility to COL, while BD Phoenix 100 system (BD, Mississauga, ON, Canada) was used to test susceptibility to other antibiotics.

## Data Availability

All data generated or analyzed as part of this study are available in the article.
